# Food for thought: the importance of nutritional well-being during COVID-19

**DOI:** 10.1017/ipm.2021.2

**Published:** 2021-01-18

**Authors:** L. Burke-Furey, F. McNicholas

**Affiliations:** 1 Department of Child & Adolescent Psychiatry, SMMS, UCD, Dublin, Ireland; 2 Paediatric Liaison Psychiatry, Children Hospital, Crumlin, Dublin 12, Ireland; 3 Child & Adolescent Psychiatry, Lucena Clinic, Rathgar Dublin 6, Ireland

**Keywords:** COVID-19, microbiome, micronutrients, nutrition, well-being

## Abstract

Individuals with mental illness have poorer physical health, nutritional status, and lowered life expectancy. Optimising their physical and nutritional status has become an increasingly important therapeutic goal. Current experience with COVID-19 has further emphasised the susceptibility to physical illness and poorer outcomes amongst individuals with mental illness and those who are nutritionally compromised. Although life as we knew it has been suspended until the widespread roll-out of a vaccine, individuals can take immediate action to improve physical and mental health by attending to and optimising their nutritional well-being. Clinicians within mental health services have a crucial role to play in assisting such change, and reminding their patients of the importance of pursuing a healthy and balanced diet.

## Introduction

Current life expectancy in Ireland, based on 2017 data, is 84 years for women and 80.4 years for men. (https://www.gov.ie/en/publication/f1bb64-health-in-ireland-key-trends-2019/accessed August 29 2020). However, increased longevity has not necessarily led to increased quality of life for all. The cost of longevity brings about an increased incidence of, and time lived with, chronic illnesses such as cancer, diabetes, cardiovascular disease, cognitive, and mental health disorders. Furthermore, disparities exist and those with severe mental illness have a lowered life expectancy by 10–20 years compared to the general population, attributable to poorer diet, lack of physical activity, smoking, and substance abuse (Ringen *et al*. 2014). The independent adverse role of psychotropic medication on weight and nutritional status is also recognised as a factor (Mazereel. *et al*. 2020). Early and significant weight gain following the introduction of the first or second-generation antipsychotics is well recognised and thought to be due to increased central appetite (Foley & Morley, 2011). Weight gain is also associated with mood stabilisers (Sachs *et al*. 2006) and antidepressants (Serretti & Mandelli, 2010), and patients with lower BMI at baseline are not immune (Gebhardt *et al*. 2009). Morbidity and mortality in psychotropic medication users are further increased by the development of dyslipidemia and insulin resistance, both strongly related to medication-induced weight changes (Abosi *et al*. 2018).

Profound lifestyle changes have occurred over the recent years with industrialisation and technological advances, with more office-based, non-manual labour, longer commutes, and lower population-level energy expenditure related to work. More and more leisure time is spent on smart devices, computers, and watching television. A recent report by the World Health Organization concluded that 80% of children aged between 11 and 17 were not getting enough physical activity (Guthold *et al*. 2020). People with severe mental illness, especially those with schizophrenia and depression, are also known to be more sedentary than their healthy counterparts (Bort-Roig *et al.* 2020; Dipasquale *et al.* 2013). There is a clear relationship between a more sedentary lifestyle, for both youth and old, and weight gain, despite clinician’s call to action (Manson *et al*. 2004). A recent Department of Health survey, from a representative sample of 7413 people living in Ireland, suggests that just over a third of adults, (37%) were of normal weight (http://tinyurl.com/yxuwotvu). Official OECD estimates place Ireland mid-table by international comparisons. Rates of obesity in children and adolescents have also increased. Indeed, the genesis of adult obesity is in childhood, and carries with it many risk factors for subsequent adult ill health, most notably cardiovascular, cancer, and diabetes. Poor dietary and activity habits established in childhood become engrained. Given that up to 60% of individuals with severe mental illness also have obesity, the additional medication-related dyslipidemia and insulin resistance, places them at additional cardiovascular risk (Abosi *et al*. 2018).

Despite being overfed, individuals with obesity have nutritional deficits in micronutrients, recognised to have anti-inflammatory, antioxidant, and antiviral properties, and have raised inflammatory markers such as C-reactive protein and fibrinogen, making them prone to infections and autoimmune disorders. Deficiencies in vitamins such as A, C, D, and E, along with lower levels of iron and zinc have also been reported. Consumption of high levels of fructose syrup, regulated by many European countries, has specifically been associated with lower levels of vitamin D and metabolic syndrome, even in the absence of increased body weight (Ferder & Inserra, 2010).

Vitamin D has been implicated in psychiatric illness for some time and receptors are prevalent in many parts of the brain associated with mental illness (Cuomo *et al*. 2019). Vitamin D is considered neuroprotective, reducing oxidative stress, modulating neuronal excitability via intracellular calcium homeostasis, and stimulating nerve growth. Low vitamin D levels have been associated with many childhood and adult disorders, such as mood disorders, psychosis, and autism, and might explain the seasonality of mood, with recommendations to monitor vitamin D levels in all psychiatric cohorts, especially the elderly, who are most at risk (Cuomo *et al*. 2019). Given the treatments of many patients with neuroleptic medication and the known associated risk of metabolic syndrome, this represents an increased risk. The association of low levels of vitamin D and the metabolic syndrome (Prasad & Kochhar, 2016) is of particular concern in a cohort of patients exposed to neuroleptic medications, which carry their own similar risk profile.

## Western diet

The increase in obesity has been attributed to the increased consumption of a Western diet, and there has been a steady and consistent shift from fresh home-grown and home-cooked foods to a diet high in fat, salt, and sugar content. Up to 15% of calories consumed are from ‘added’ sugars, often in the form of fructose syrup. These sugars are viewed as ‘empty calories’, with minimum essential nutrients or dietary fibre, leading to overweight but undernourished individuals. These highly processed foods, additive-rich but nutritionally poor, are often cheap, palatable, and easily stored with long shelf lives, making them popular choices in the lower socio-economic classes or those with poorer adaptive daily living skills. Poor dietary choices and resultant higher BMIs, in those economically more disadvantaged, may well be driven by lower cost; it is also influenced by other lifestyle habits such as smoking and excess alcohol consumption and lower physical activity, behaviours also found in those with severe mental illness (Bort-Roig *et al*. 2020; Dipasquale *et al.* 2013) . Furthermore, the co-occurrence of depression in females provided the main pathway linking lower SES and BMI (Beydoun & Wang, 2010). In contrast, a healthy diet consists of the ingestion of a wide variety of fresh fish, fruit, and vegetable, nuts, and unsaturated fats, giving a balance of essential fats, fibre, and macronutrients, and consumed at a rate commensurate with energy expenditure. Poorer planning and functioning in activities of daily living, more prevalent in individuals with mental illness, might pose an additional impediment to home cooking and sourcing of foods, and promote the adoption of a Western-style diet, and consumption of higher proportions of processed foods.

The type and diversity of food eaten also shapes, and is driven by, millions of microorganisms lining our digestive tract. These microbes in turn can lead to food preferences, and independent of calories consumed, alterations in body weight, blood sugar, and cholesterol (Patterson *et al*. 2016). Both beneficial and harmful microbes exist, playing a role in inducing cytokines and other anti or pro-inflammatory/immunity cells, synthesising certain vitamins and neurotransmitters, and playing a role as part of the larger gut–brain axis. The end result is an individually specific microbiome either promoting or hindering physical, cognitive, and mental health.

## Relationship of diet and mental health

It is, therefore, not surprising that a consistent relationship between the quality of diet and health in adults has been found, over and above other potential confounders of social-economic class, education, and even BMI (O’Neil *et al*. 2014). These apply to both physical and mental health. Cross-sectional and prospective studies show a relationship between baseline quality of diet and subsequent depression, with poorer diet at baseline predicting poorer mental health and quality of life, and a protective effect linked to good baseline diet (Jacka *et al*. 2011). There was no evidence of reverse causality, in that poorer mental health did not shift dietary habits. This is also true in the adolescent population, highlighting the long-term impact of early dietary choices and the potential preventative role of nutritional advice and education.

A systematic review concludes that changing from a Western-style diet to a traditional Mediterranean diet confers an advantage against depression (Lassale *et al*. 2019). Such is the evidence base in depression, that omega-3-polyunsaturated fatty acids, or Ω-3-PUFA, are recommended in international guidelines for the treatment of depression in adults, children, pregnant women, and the elderly (Guu *et al*. 2019). The recognised beneficial effect of removing additives and food colourings in diets of youth with ADHD has been expanded to youth with other behavioural difficulties (Stevenson, 2010), with other recent studies questioning a causal role of the Western diet in ADHD development (Ríos-Hernández *et al.* 2017).

With nutritional modifications becoming part of mainstream treatments, the industry is also shifting from exclusively focussing on new drugs towards a search for nutritional supplements and the healthy food industry has become lucrative. Regrettably, many ‘miracle-products’ are just that, with little evidence, benefits and indeed if not carefully monitored, at risk of adverse effects. At the same time, advances in nutritional science indicate that optimal intake levels of vitamins, minerals, and fibre, and adherence to a Mediterranean diet, may protect against these long-term diseases.

Personal behavioural change also plays a major part. In the Irish Survey of Lifestyle Attitudes and Nutrition (SLAN) completed by 10,364 adults, the health-related effects of adopting various healthier lifestyle choices showed that those who were able to incorporate four such behaviours (being physically active, consuming five or more fruit and vegetable servings daily, not smoking, and moderate alcohol intake) were significantly more likely to report better general and mental health (Harrington *et al*. 2010). Despite health-related behavioural changes being recognised as difficult in adolescents, it is never too early or too late to endeavour to facilitate a positive motivating environment, targeting family, peers, and social networks, initiated from the clinicians’ office.

## COVID-19 and nutrition

Following the declaration in March 2020 by the World Health Organization of the global COVID-19 pandemic, governments across the world imposed unprecedented restrictions, shutting schools, businesses, and travel in an effort to protect society and slow down the spread of the virus. At the start of lockdown, the initial nutritional concern was that of food availability, especially for the elderly who were advised to cocoon. A shift from fresh to frozen or processed foods was feared should food trade deliveries dry up, accompanied by some anecdotal media reports of bulk buying. This did not happen and fresh food remained plentiful, as did written and video media broadcasts on how to spend lockdown experimenting with cooking. For certain groups, such as those with an eating disorder, the presence of readily available and potentially threatening food, with restrictions on exercise and outdoor access, caused intense anxiety, and deterioration in symptoms (Schlegl *et al.* 2020). For others, the opportunity to constantly ‘graze’ led to weight gain, and obesity, with all the aforementioned risks. A poorer prognostic risk in obese individuals has been suggested with COVID-19 (Alberca *et al*. 2020) and a higher rate of obesity in older adults in Italy was proposed as a putative explanation to account for the excess deaths in patients with COVID-19 when compared to China (Dietz & Santos-Burgoa, 2020). In addition, more and more evidence is emerging linking vitamin D deficiency to increased infectivity, morbidity, and mortality in COVID-19 (Aygun, 2020). Interventional studies emerged as to the beneficial effects of treatment with the active form of vitamin D, calcifediol (Castillo *et al*. 2020). Given levels of vitamin D are recognised to be low both in Ireland (Cashman *et al.* 2013), particularly now in winter months, and low amongst those with mental illness (Cuomo *et al*. 2019), this places patients attending Mental Health Services in Ireland at double jeopardy.

Level 5 restrictions, about to recommence in January 2021, will limit access to outdoor activities, team sports, and gyms, and could potentially lead to poorer physical health and weight gain. Studies that have investigated activity levels during earlier lockdowns have shown both the benefit of increased activity and the negative effect of increased sedentary time on physical and mental health (Cheval *et al*. 2020). Working from home can further increase sedentary behaviour, and unfettered access to readily available snacking foods. Limited social engagement limits mood-boosting experiences and can increase the risk of loneliness, isolation, and lead to despondency and poor self-care. Elderly persons, fearful of the resurgence of disease and those with pre-existing mental illness may be most at risk of poor nutritional care.

## Clinical assessment and nutritional advice

Scientifically robust studies in mental health have now caught up with nutritional medicine and attest to the importance of quality and diversity of diet and microbiome in physical and psychiatric illness. The greater prevalence of obesity and poorer physical health amongst those with mental illness has been well documented. Nutrition is increasingly being considered as a crucial and potentially modifiable factor in the aetiology and maintenance of many illnesses, in both young and old, and one that clinicians should incorporate into their usual practice. However, at odds with the growing evidence of the importance of a healthy and balanced dietary intake, its role in mainstream clinical practice or indeed medical training is less developed. Routine primary care clinical assessments do not typically enquire about the adequacy of dietary intake or physical activity, weight, and BMI infrequently calculated. The recognition of medication-related metabolic effects amongst patients on anti-psychotics has led to monitoring of weight in many, and pharma-sponsored healthy lifestyle programmes. The presence of a dietician or nutritionist as a core member of the multidisciplinary team is not yet common practice. Studies suggest that when such nutritional advice is offered, patients do try to follow it.

Given physical health is often poor amongst mental health services users, coupled with difficulties in self-motivation and self-care, psychiatrists could play a crucial role in reviewing and advising about healthy lifestyle choices, diet, exercise, sleep, and substance use. Given our clinical experience and familiarity with Socratic questioning and behavioural management, we are well placed to enquire about physical health and consider dietary manipulations as part of standard therapeutic work. Given the emerging data on high adverse outcomes in COVID-19 amongst those with obesity, low vitamin D levels, or mental ill health, this brings urgency for such a focus.

## Conclusion

There has been a seismic shift in the understanding of the importance of a balanced diet to our health and longevity. There is very good evidence of the role of nutritional manipulation going beyond calorie restriction and weight loss. Equally, there has been ample evidence of the negative effect of the Western-style diet on populations. As we emerge from the Christmas period, with the excess of food, drink, and more sedentary behaviour, and as we enter the period of new resolutions, clinicians are well poised to assist in behavioural change.

Each consultation offers the clinician the opportunity to enquire about the quality of dietary intake, and advise patients to incorporate freshly cooked fruits and vegetables as part of standard daily intake, limit the amount of processed fast foods consumed and take a daily walk to search perhaps for the Irish sun. Supplementation with vitamin D is particularly important during the winter months. It may well be that behind this COVID-19 cloud, better eating and lifestyle approaches will shine through, and not a moment too soon.

Suggested advice a clinician might give to their patients during the consultation is listed in Tables 1 and 2. A more detailed leaflet is available from the authors upon request.

**Table 1.** Optimising nutrition and well-being

1. Health screening with GP	Check blood for excess and deficiencies.
2. Reduce stress	Slow down and connect with your breath. Yoga and meditation. Gratitude. Mindful eating (chew each mouthful 20 times). Complimentary therapies (kinesiology, acupuncture, massage).
3. Reduce toxicity	Sugar. Alcohol. Smoking. Food intolerances. Caffeine. Viruses, harmful bacteria, parasites, fungus, heavy metals. Environmental toxins. Toxic relationships.
4. Eat a nutritious varied diet (to increase gut health, balance blood sugars and hormones)	Carbohydrates. Proteins. Healthy fats. Good gut bacteria. Water (2 l). Vitamins, minerals, enzymes, salts. Colour matters, eat with your eyes. Combine a healthy carbohydrate with either a protein or healthy fat to help balance blood sugars. Six weeks to balance blood sugars and hormones.
5. Consider supplements and use essential oils	Probiotics. Vitamin D. Vitamin C. Zinc. Omega-3 fatty acids. Essential oils.
6. Adequate exercise	Aim for at least 30 minutes of aerobic exercise daily. Aim for 10,000 steps daily. Get outdoors in nature and sunshine.
7. Adequate sleep	Aim for 8 hours. Reduce technology at night.
8. Maintain a healthy weight	Waist measurements and BMI matter. Portion size matters. Visualise your dinner plate. Aim to eat within a 12-hour window.

**Table 2.** Recommended reading for patients

**Recommended reading for patients** The Four Pillar Plan (Relax, Eat, Move, Sleep) by Dr Rhangan Chattergee. The Gut Makeover (4 Weeks To Nourish Your Gut, Revolutionise Your Health & Lose Weight) by Jeannette Hyde. The Happy Kitchen (Good Mood Food) by Rachel Kelly. The Happy Pear Vegan Cooking For Everyone by David and Stephen Flynn. Quick & Easy Plant-Based Deliciousness by Deliciously Ella. Gut Feeling (Delicious Low Fodmap Recipes To Soothe The Symptoms Of A Sensitive Gut) by Lorraine Maher and Paul Mee. Delete if there are too much word count. In Praise of Walking (The New Science of How We Walk & Why It’s Good For Us) by Shane O’Mara. Why We Sleep by Matthew Walker. Brain Changer (How Diet Can Save Your Mental Health) by Professor Felice Jacka. Why Isn’t My Brain Working (A Revolutionary Understanding Of Brain Decline And Effective Strategies To Recover Your Brain Health) by Dr Datis Kharrazian. The Psychobiotic Revolution (Mood, Food And The New Science Of The Gut-Brain Connection) by John F. Cryan, Scott C. Anderson and Ted Dinan.

## References

[ref1] Abosi O , Lopes S , Schmitz S , Fiedorowicz JG (2018). Cardiometabolic effects of psychotropic medications. Hormone Molecular Biology and Clinical Investigation 36, 20170065. doi: 10.1515/hmbci-2017-0065 PMC6818518 29320364

[ref2] Alberca RW , Oliveira LDM , Branco ACCC , Pereira NZ , Sato MN (2020). Obesity as a risk factor for COVID-19: an overview. Critical Reviews in Food Science and Nutrition 1–15. 10.1080/10408398.2020.1775546 32539446

[ref3] Aygun H (2020). Vitamin D can prevent COVID-19 infection-induced multiple organ damage. Naunyn-schmiedeberg’s Archives of Pharmacology 25, 1–4. 10.1007/s00210-020-01911-4 PMC7246956 32451597

[ref4] Beydoun MA , Wang Y (2010). Pathways linking socioeconomic status to obesity through depression and lifestyle factors among young US adults. Journal of Affective Disorders 123, 52–63. 19853306 10.1016/j.jad.2009.09.021 PMC2860015

[ref5] Bort-Roig J , Briones-Buixassa L , Felez-Nobrega M , Guàrdia-Sancho A , Sitjà-Rabert M , Puig-Ribera A (2020). Sedentary behaviour associations with health outcomes in people with severe mental illness: a systematic review. European Journal of Public Health 30, 150–157. 30793737 10.1093/eurpub/ckz016

[ref6] Cashman KD , Muldowney S , McNulty B , Nugent A , FitzGerald AP , Kiely M , Flynn A (2013). Vitamin D status of Irish adults: findings from the National Adult Nutrition Survey. British Journal of Nutrition 109, 1248–1256. 10.1017/S0007114512003212 22883239

[ref7] Castillo ME , Costa LME , Barrios JMV , Díaz JFA , Miranda JL , Bouillon R , Gomez JMQ (2020). Effect of calcifediol treatment and best available therapy versus best available therapy on intensive care unit admission and mortality among patients hospitalized for COVID-19: a pilot randomized clinical study. The Journal of Steroid Biochemistry and Molecular Biology 203, 105751. 32871238 10.1016/j.jsbmb.2020.105751 PMC7456194

[ref8] Cheval B , Sivaramakrishnan H , Maltagliati S , Fessler L , Forestier C , Sarrazin P , Orsholits D , Chalabaev A , Sander D , Ntoumanis N , Boisgontier MP (2020). Relationships between changes in self-reported physical activity, sedentary behaviour and health during the coronavirus (COVID-19) pandemic in France and Switzerland. Journal of Sports Sciences 1–6. 10.1080/02640414.2020.1841396 33118469

[ref9] Cuomo A , Maina G , Bolognesi S , Rosso G , Beccarini Crescenzi B , Zanobini F , Goracci A , Facchi E , Favaretto E , Baldini I , Santucci A (2019). Prevalence and correlates of vitamin D deficiency in a sample of 290 inpatients with mental illness. Frontiers in Psychiatry 10, 167. 31001150 10.3389/fpsyt.2019.00167 PMC6455075

[ref10] Dietz W , Santos-Burgoa C (2020). Obesity and its Implications for COVID-19 Mortality. Obesity 28, 1005–1004. 32237206 10.1002/oby.22818

[ref11] Dipasquale S , Pariante CM , Dazzan P , Aguglia E , McGuire P , Mondelli V (2013). The dietary pattern of patients with schizophrenia: a systematic review. Journal of Psychiatric Research 47, 197–207. 23153955 10.1016/j.jpsychires.2012.10.005

[ref12] Ferder L , Inserra F (2010). The role of high-fructose corn syrup in metabolic syndrome and hypertension. Current Hypertension Reports 12, 105–112. 20424937 10.1007/s11906-010-0097-3

[ref13] Foley DL , Morley KI (2011). Systematic review of early cardio-metabolic outcomes of the first treated episode of psychosis. Archives of General Psychiatry 68, 609–616. 21300937 10.1001/archgenpsychiatry.2011.2

[ref14] Gebhardt S , Haberhausen M , Heinzel-Gutenbrunner M , Gebhardt N , Remschmidt H , Krieg JC , Hebebrand J , Theisen FM (2009). Antipsychotic-induced body weight gain: predictors and a systematic categorization of the long-term weight course. Journal of Psychiatric Research 43, 620–626. 19110264 10.1016/j.jpsychires.2008.11.001

[ref15] Guthold R , Stevens GA , Riley LM , Bull FC (2020). Global trends in insufficient physical activity among adolescents: a pooled analysis of 298 population-based surveys with 1·6 million participants. The Lancet Child & Adolescent Health 4, 23–35. 31761562 10.1016/S2352-4642(19)30323-2 PMC6919336

[ref16] Guu TW , Mischoulon D , Sarris J , Hibbeln J , McNamara RK , Hamazaki K , Freeman MP , Maes M , Matsuoka YJ , Belmaker RH , Jacka F (2019). International Society for Nutritional Psychiatry Research Practice Guidelines for Omega-3 fatty acids in the treatment of major depressive disorder. Psychotherapy and Psychosomatics 88, 263–73. 31480057 10.1159/000502652

[ref17] Harrington J , Perry IJ , Lutomski J , Fitzgerald AP , Shiely F , McGee H , Barry MM , Van Lente E , Morgan K , Shelley E (2010). Living longer and feeling better: healthy lifestyle, self-rated health, obesity and depression in Ireland. European Journal of Public Health 20, 91–95. 19587230 10.1093/eurpub/ckp102

[ref18] Jacka FN , Kremer PJ , Berk M , de Silva-Sanigorski AM , Moodie M , Leslie ER , Pasco JA , Swinburn BA (2011). A prospective study of diet quality and mental health in adolescents. PloS one 6, e24805. 21957462 10.1371/journal.pone.0024805 PMC3177848

[ref19] Lassale C , Batty GD , Baghdadli A , Jacka F , Sánchez-Villegas A , Kivimäki M , Akbaraly T (2019). Healthy dietary indices and risk of depressive outcomes: a systematic review and meta-analysis of observational studies. Molecular Psychiatry 24, 965–986. 30254236 10.1038/s41380-018-0237-8 PMC6755986

[ref20] Manson JE , Skerrett PJ , Greenland P , VanItallie TB (2004). The escalating pandemics of obesity and sedentary lifestyle: a call to action for clinicians. Archives of Internal Medicine 164, 249–258. 14769621 10.1001/archinte.164.3.249

[ref21] Mazereel V , Detraux J , Vancampfort D , Van Winkel R , De Hert M (2020). Impact of psychotropic medication effects on obesity and the metabolic syndrome in people with serious mental illness. Frontiers in Endocrinology 11. 10.3389/fendo.2020.573479 PMC7581736 33162935

[ref22] O’neil A , Quirk SE , Housden S , Brennan SL , Williams LJ , Pasco JA , Berk M , Jacka FN (2014). Relationship between diet and mental health in children and adolescents: a systematic review. American Journal of Public Health 104, e31–e42. 10.2105/AJPH.2014.302110 PMC4167107 25208008

[ref23] Patterson E , Ryan PM , Cryan JF , Dinan TG , Ross RP , Fitzgerald GF , Stanton C (2016). Gut microbiota, obesity and diabetes. Postgraduate Medical Journal 92, 286–300. 26912499 10.1136/postgradmedj-2015-133285

[ref24] Prasad P , Kochhar A (2016). Interplay of vitamin D and metabolic syndrome: a review. Diabetes & Metabolic Syndrome: Clinical Research & Reviews 10, 105–112. 10.1016/j.dsx.2015.02.014 25813139

[ref25] Ringen PA , Engh JA , Birkenaes AB , Dieset I , Andreassen OA (2014). Increased mortality in schizophrenia due to cardiovascular disease–a non-systematic review of epidemiology, possible causes, and interventions. Frontiers in Psychiatry 5, 137. 25309466 10.3389/fpsyt.2014.00137 PMC4175996

[ref26] Ríos-Hernández A , Alda JA , Farran-Codina A , Ferreira-García E , Izquierdo-Pulido M (2017). The Mediterranean diet and ADHD in children and adolescents. Pediatrics 139. 10.1542/peds.2016-2027 28138007

[ref27] Sachs G , Bowden C , Calabrese JR , Ketter T , Thompson T , White R , Bentley B (2006). Effects of lamotrigine and lithium on body weight during maintenance treatment of bipolar I disorder. Bipolar Disorders 8, 175–181. 16542188 10.1111/j.1399-5618.2006.00308.x

[ref28] Schlegl S , Maier J , Meule A , Voderholzer U (2020). Eating disorders in times of the COVID-19 pandemic—Results from an online survey of patients with anorexia nervosa. International Journal of Eating Disorders. 10.1002/eat.23374 PMC7461418 32841413

[ref29] Serretti A , Mandelli L (2010). Antidepressants and body weight: a comprehensive review and meta-analysis. The Journal of Clinical Psychiatry 71, 1259–1272. 21062615 10.4088/JCP.09r05346blu

[ref30] Stevenson J (2010). Recent research on food additives: Implications for CAMH. Child and Adolescent Mental Health 15, 130–133. 32847234 10.1111/j.1475-3588.2010.00563.x

[ref31] https://www.hse.ie/eng/about/who/healthwellbeing/our-priority-programmes/heal/heal-docs/healthy-ireland-survey-2018-summary-of-findings.pdf Accessed 20 September 2020.

